# Cognitive intervention through a training program for picture book reading in community-dwelling older adults: a randomized controlled trial

**DOI:** 10.1186/1471-2318-14-122

**Published:** 2014-11-21

**Authors:** Hiroyuki Suzuki, Masataka Kuraoka, Masashi Yasunaga, Kumiko Nonaka, Ryota Sakurai, Rumi Takeuchi, Yoh Murayama, Hiromi Ohba, Yoshinori Fujiwara

**Affiliations:** Research Team for Social Participation and Community Health, Tokyo Metropolitan Institute of Gerontology, 35-2 Sakae-cho, Itabashi-ku, Tokyo, Japan

**Keywords:** Cognitive intervention, Community-dwelling older adults, Picture book reading, Mild cognitive impairment, Randomized controlled trial

## Abstract

**Background:**

Non-pharmacological interventions are expected to be important strategies for reducing the age-adjusted prevalence of senile dementia, considering that complete medical treatment for cognitive decline has not yet been developed. From the viewpoint of long-term continuity of activity, it is necessary to develop various cognitive stimulating programs. The aim of this study is to examine the effectiveness of a cognitive intervention through a training program for picture book reading for community-dwelling older adults.

**Methods:**

Fifty-eight Japanese older participants were divided into the intervention and control groups using simple randomization (n =29 vs 29). In the intervention group, participants took part in a program aimed at learning and mastering methods of picture book reading as a form of cognitive training intervention. The control group listened to lectures about elderly health maintenance. Cognitive tests were conducted individually before and after the programs.

**Results:**

The rate of memory retention, computed by dividing Logical Memory delayed recall by immediate recall, showed a significant interaction (p < .05) in analysis of covariance. Simple main effects showed that the rate of memory retention of the intervention group improved after the program completion (p < .05). In the participants with mild cognitive impairment (MCI) examined by Japanese version of the Montreal Cognitive Assessment (MoCA-J) (n =14 vs 15), significant interactions were seen in Trail Making Test-A (p < .01), Trail Making Test-B (p < .05), Kana pick-out test (p < .05) and the Mini-Mental State Examination (p < .05).

**Conclusions:**

The intervention effect was found in delayed verbal memory. This program is also effective for improving attention and executive function in those with MCI. The short-term interventional findings suggest that this program might contribute to preventing a decline in memory and executive function.

**Trial registration:**

UMIN-CTR: UMIN000014712 (Date of ICMJE and WHO compliant trial information disclosure: 30 July 2014)

## Background

Dementia is a topic of international concern, with rapid increase in the number of older adults with dementia in many countries. As the total worldwide societal cost of dementia, based on a dementia population of 34.4 million persons with dementia, was estimated to $422 billion in 2009, including $142 billion for informal care (34%), the increase of people with cognitive decline causes huge economic and societal costs and becomes a burden to the community [1].

On the other hand, recent studies reported that the age-adjusted prevalence of senile dementia in the 2000s tended to be falling as compared with that of the 1980s [2, 3]. Non-pharmacological interventions are expected to be an important strategy for reducing age-adjusted prevalence of senile dementia, considering that complete medical treatment for cognitive decline has not yet been developed [4]. Previous randomized controlled trials of community-dwelling older adults have found that physical exercise programs contribute to improvement in cognitive functions [5, 6]. Aerobic exercise training is effective at reversing hippocampal volume loss in late adulthood, which is accompanied by improved memory function [5]. A 6-month program of physical activity provided a modest improvement in cognition over an 18-month follow-up period for adults with subjective memory impairment [6].While there is strong evidence that physical exercise intervention is effective for preventing cognitive function decline, experiencing pain or suffering from injury due to exercise makes it more difficult for not a few older adults to continue physical exercise.

For mentally-normal older adults, continuation of activity is easier for intellectual activities than physical exercises. Age-related decline of cognitive functions advances over a long period of time [7], so continuous stimulating activities are important. The cognitive reserve hypothesis [8, 9] assumes that frequent use of the brain neural network strengthens its connections and helps to prevent possible morbid invasions. Based on this idea, it is expected that a decline in cognitive functions may be delayed both by performing intellectual activities and by communicating with others.

In addition, age-related impairments of memory are thought to originate from the lack of hippocampal activation during memory encoding. When young people memorize novel information, cerebral blood flow of the hippocampus, a region of the brain that participates in memory, increases. On the other hand, when older adults do the same work, the cerebral blood flow of the hippocampus does not increase [10]. It is important to acquire memorization strategies in order to compensate for the age-related decline in hippocampal activation. From the viewpoint of long-term continuity of activity, it is necessary to develop various intellectual programs that prevent the decline of cognitive functions.

Therefore, this study focused on senior volunteers who read picture books to children. Reading picture books aloud to children encompasses high intellectual activity, and it includes additional benefits of the creation and maintenance of relationships with others [11]. Reading picture books to children is popular among schools in Japan. In addition, lifelong learning programs for mastering the skills for reading aloud to children have been popular among middle and old-aged people in communities. The intellectual activity by, for example, picture book reading is effective for preserving IADL among active older adults [12]. Furthermore, it is reasonable and suitable to engage longer in such kinds of volunteer activity for older adults, even if they have lower physical function.

As this activity showed various potential, we developed a cognitive intervention program based on acquiring picture book reading skills with the aim of understanding the effects of such an intervention on cognitive decline in older adults. The program including this advanced intellectual activity offers many opportunities to memorize and tell stories. Therefore, we expect an improvement in verbal memory. In this study we conducted a randomized controlled trial to explore the effectiveness of cognitive intervention through a training program for picture book reading in community-dwelling older adults.

## Methods

### Study design and participants

**Figure 1:**
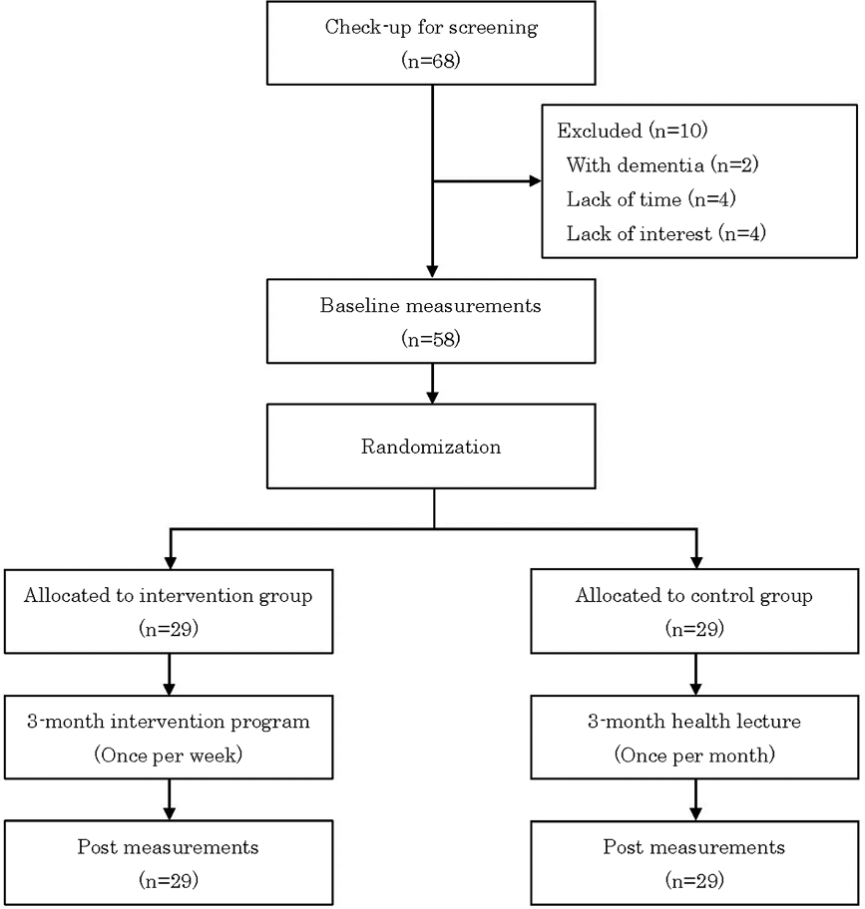
**CONSORT flow diagram.**

The present study employed a randomized controlled trial design in urban areas of Tokyo. We advertised the research program through community newspapers or newsletters. We recruited community-dwelling older adults based on the following criteria: (i) age 65 years and older; (ii) worried about memory problems; (iii) absence of self-reported dementia. Figure 1 describes the CONSORT flow diagram of this study. Sixty-eight applicants participated in the initial check-up for screening. After the 68 applicants were screened by a questionnaire and medical interview, a geriatrician who specialized in dementia diagnosed two persons as having dementia according to the criteria of International Statistical Classification of Diseases and Related Health Problems 10th Revision. No applicant had other neurological impairments including mental retardation. Eight persons did not participate in baseline measurement due to scheduling conflicts or lack of interest. Therefore, 58 older adults who offered participation were divided into the intervention and control groups using simple randomization. All participants provided written informed consent to participate in the study. Anonymity and confidentiality were also preserved at all times and the principles of the Declaration of Helsinki were respected. The study design and protocol were approved by the Institutional Review Board and Ethics Committee of the Tokyo Metropolitan Institute of Gerontology (Acceptance No. 14, 1, 2010).

### Procedure

Baseline measurement and cognitive tests were conducted on each participant individually. After randomly dividing the participants into two groups, the intervention group started the picture book reading program. The control group participated in lectures about elderly health maintenance once a month (three times in total). After the 12-week picture book reading program had been completed, follow-up cognitive tests were conducted for the participants of both groups. The follow-up measurements were recorded by examiners blinded to the allocation. In order to maintain fairness to all participants, after the end of the follow-up tests, the control group also undertook the picture book training program.

### Intervention program

Participants took part in a program aimed at learning and mastering methods of picture book reading as a form of cognitive training intervention. This program was based on the pilot study REPRINTS (Research of Productivity by Intergenerational Sympathy) [11], which engages senior volunteers in picture book reading to children. Fujiwara et al. reported that self-rated health improved in older adults who volunteered in the program [11]. Picture book reading has a salutary effect not only on self-rated health but also on cognitive function. The reason is that the majority of activities which stimulate cognitive functions are included in the practice of picture book reading. In order to read while showing the pictures in a story to children, it is necessary to memorize the outline of the story. The executive function of reading a book within an assigned time is also needed. Based on these ideas, we created a curriculum which specialized in cognitive function training by means of picture book reading. Participants learned how to choose a book, how to memorize a tale, how to show a picture, how to enunciate, and how to read with feeling. Since referencing to the self when encoding information is a powerful memory strategy [13, 14], the empathy to stories was emphasized in this program.

The participants were required to perform as if they were actually reading to children. In addition to the research staff, the program was managed by an instructor skilled at picture book reading. The individual presentation of picture book reading was held in the middle of the program. In the final phase, a group exhibition by a small number of people was held. The program was held once a week, over a twelve-week period. One training session lasted approximately two hours.

### Measurements

The following tests were conducted to evaluate each cognitive domain: Logical Memory (LM) I and II subscale from Wechsler Memory Scale Revised (WMS-R) [15, 16]; subtests of Wechsler Adult Intelligence Scale-III [17], such as Digit Span Forward (DSF) and Digit Span Backward (DSB); Trail Making Test (TMT) Part A and Part B [18]; *Kana* Pick-out Test (KPT) [19]; and verbal fluency tests [20]. In addition, the following assessments were applied to measure global cognitive functions: the Mini-Mental State Examination (MMSE) [21], and the Japanese version of the Montreal Cognitive Assessment (MoCA-J) [22].

LM I and II are story recall tests that assess immediate and delayed verbal memory. LM I is an assessment of immediate memory. In this test, the examiner tells the first story and participants are asked to recall the contents of the story immediately. Then the examiner tells the second story and participants recall the story. LM II is an assessment of delayed memory; the participants were asked to recall the two stories thirty minutes after the stories were read. Scores were calculated by adding the elements of the story that were recalled. The maximum score for both immediate and delayed recall is 50 points. Higher scores indicate better memory function. LM II was the primary outcome indicator in this study, since story recall tests directly reflect everyday memory [23]. Also, the DSF, which measures simple memory span, and the DSB, which measures working memory capacity, were used as other memory indicators.

The Trail Making Test-A (TMT-A), Trail Making Test-B (TMT-B) and KPT were conducted for evaluation of executive function. Both versions of the TMT consisted of 25 circles scattered over a sheet of paper. In TMT-A, the circles were numbered 1 to 25, and the participants were required to draw lines to connect the numbers in ascending order and the time was measured. In TMT-B, the circles included either numbers or letters. The numbers used were 1 to 13 and the letters used were the first 12 letters of the Japanese *Hiragana* alphabet. The participants were required to connect the numbers and letters alternately. Fewer seconds on TMT-A and -B indicate better attention and executive function. The test-retest reliability for the Japanese version of TMT-B was 0.595 [24]. The KPT, developed in Japan, consists of a short story written in Japanese *Hiragana* characters. Participants are required to read and understand the story in two minutes and to find as many vowel letters as possible. The participants circle the appropriate letters while reading the story. In this test, verbal executive function is measured. The story consists of 406 letters, and 61 vowels are included. The test is scored by counting letters which are circled correctly within two minutes. In this test, higher scores indicate better verbal executive function.

Verbal fluency tests in which participants are asked to generate as many words as possible according to prescribed cues within a minute were used as verbal function measurement. Phonemic fluency was assessed using the letter “ka” and semantic fluency was assessed using the category “animals”. The total number of words appropriately generated was considered the score.

The MMSE is included as a general cognitive assessment with a maximal score of 30 and is often used as a screening for dementia. The MoCA [25] is a brief cognitive screening tool for older adults with mild cognitive impairment (MCI). On a clinical basis, 25/26 is used as a cut-off score in the detection of MCI, and the same cut-off score is used in the Japanese version [22]. The MoCA is gaining credibility due to sensitively identifying MCI, and decreasing susceptibility to cultural and educational biases [26]. This study considered the participants whose score on the MoCA-J at baseline was less than 26 points to have MCI. The Geriatric Depression Scale short form (GDS-15) [27] and the Tokyo Metropolitan Institute of Gerontology-Index of Competence (TMIG-IC) [28] were measured in the baseline inspection. The TMIG-IC consisted of 13 multidimensional items classified under three subscales of instrumental self-maintenance, intellectual activity and social role. The cases which had a defect in inspection implementation were excluded from analysis.

### Data analysis

Baseline demographics between the intervention and the control groups were compared using t-tests and a chi-square test. Differences in cognitive tests among groups over time were computed using a series of mixed model analysis of covariance (ANCOVA). Group assignment (intervention group or control group) was the fixed factor and assessment time (pre and post) was the repeated measures factor. Age and education were considered as covariates. η^2^ was used as an estimate of the effect size. After finding a significant interaction in ANCOVA, we performed tests of simple main effects to show the effects of the intervention.

## Results

### Characteristics of the participants

Table 1 shows the characteristics of the intervention group (N =29) and the control group (N =29). There were no significant differences observed between the two groups in age, gender, education level, scores of MMSE, MoCA-J, GDS, and TMIG-IC at baseline.

**Table 1:** **Demographic at baseline measurements in both intervention and control groups**

		Intervention group ***N*** = 29			Control group ***N*** = 29			***P***Value ^1^
Age (mean ± SD)	Years	73.0 ± 7.1			73.3 ± 5.4			0.853
Gender (Female/Male)	*N*	27 / 2			26 / 3			0.640
Education (mean ± SD)	Years	12.6 ± 2.0			13.1 ± 2.5			0.318
MMSE (mean ± SD)	Score(0–30)	27.1 ± 1.7			26.6 ± 2.2			0.318
MoCA-J (mean ± SD)	Score(0–30)	25.2 ± 3.2			24.1 ± 3.7			0.203
GDS-15 (mean ± SD)	Score(0–15)	4.1 ± 2.5			3.5 ± 3.1			0.405
TMIG-IC								
Total score (mean ± SD)	Score(0–13)	10.9 ± 1.3			10.8 ± 1.7			0.861
Instrumental self-maintenance (mean ± SD)	Score(0–5)	5.0 ± 0.0			5.0 ± 0.2			0.322
Intellectual activity (mean ± SD)	Score(0–4)	3.1 ± 1.0			3.1 ± 1.2			0.905
Social role (mean ± SD)	Score(0–4)	3.8 ± 0.6			3.8 ± 0.6			1.000

MMSE: Mini-Mental State Examination; MoCA-J: Japanese version of the Montreal Cognitive Assessment; GDS-15: Geriatric Depression Scale short form; SD: Standard Deviation; TMIG: Tokyo metropolitan institute of gerontology-Index of Competence.

^1^The chi-square test was used to test association among categorical variables, and *t*-tests was used to compare the means of continuous variables.

### Analysis of effects of intervention

Scores of the cognitive tests in each group appear in Table 2. In the LM I, neither interaction nor significant main effect of group or time were found. However, in the LM II, which is an evaluation of delayed memory, a significant interaction between group and time (F (1, 54) =6.97, p =0.011, η^2^ = 0.103) was found. Simple main effects showed that the score of the intervention group improved after the program completion (p <0.001). Since the LM II was the primary outcome indicator of this study, the intervention effect was analyzed in more detail. The rate of memory retention was computed by dividing delayed recall score by immediate recall score, and this was set to the Δ LM. Mean ± standard deviations of the actual retention rate of the memory recall test were 62.7 ± 27.5% at pre-program and 74.0 ± 26.0% at post-program in the intervention group. Those of the control group were 58.8 ± 29.5% at pre-program and 56.7 ± 28.0% at post-program. In the Δ LM, the same significant interaction between group and time (F (1, 54) =4.74, p =0.034, η^2^ = 0.081) was found. Simple main effects showed that the rate of retention of the intervention group improved after the program completion (p =0.012). There were no significant differences in the DSF and the DSB which were the remaining memory indicators.

**Table 2:** **Scores of cognitive tests at pre and post program in both intervention and control groups**

		Intervention group (***N*** = 29)						Control group (***N*** = 29)						ANCOVA ^1^		
		Pre			Post			Pre			Post			***F***	***P***value ^2^	Effect size ^2,3^
		Mean ± SD			Mean ± SD			Mean ± SD			Mean ± SD					
MMSE	Score (0–30)	27.1 ± 1.7			28.0 ± 1.6			26.6 ± 2.2			27.0 ± 2.2			1.17	0.284	0.021
MoCA-J	Score (0–30)	25.2 ± 3.2			25.4 ± 3.1			24.1 ± 3.7			24.6 ± 3.2			0.31	0.583	0.005
Logical memory I (immediate)	Score (0–50)	19.2 ± 6.2			22.0 ± 7.4			16.5 ± 6.7			19.3 ± 7.0			0.03	0.873	0.000
Logical memory II (delayed)	Score (0–50)	13.1 ± 7.1			17.3 ± 8.8			10.9 ± 7.9			12.3 ± 8.4			6.97	0.011	0.103
∆ Logical memory^4^ (delayed / immediate)	Rate of retention (%)	62.7 ± 27.5			74.0 ± 26.0			58.8 ± 29.5			56.7 ± 28.0			4.74	0.034	0.220
Trail making test, part A	Seconds to completion	48.1 ± 16.3			43.6 ± 19.2			51.5 ± 18.1			55.2 ± 27.1			2.63	0.111	0.046
Trail making test, part B	Seconds to completion	152.6 ± 111.3			140.0 ± 86.8			142.1 ± 60.3			174.5 ± 132.0			2.83	0.098	0.051
Kana pick-out test	Score (0–61)	29.1 ± 10.3			32.2 ± 10.2			27.1 ± 10.8			27.8 ± 11.5			2.86	0.097	0.049
Letter fluency, "ka"	Number of words	11.0 ± 3.3			12.3 ± 4.0			10.5 ± 3.9			11.4 ± 3.0			0.03	0.872	0.000
Category Fluency, “Animal”	Number of words	16.5 ± 4.7			16.6 ± 4.2			15.8 ± 4.1			14.5 ± 4.3			2.19	0.144	0.035
WAIS-III																
Digit span forward	Raw score (0–16)	8.9 ± 2.3			8.8 ± 2.2			9.0 ± 2.1			9.2 ± 1.8			0.52	0.474	0.009
Digit span backward	Raw score (0–14)	5.5 ± 1.1			5.6 ± 1.7			5.5 ± 1.6			5.7 ± 2.1			0.10	0.750	0.002

MMSE: Mini-Mental State Examination; MoCA-J: Japanese version of the Montreal Cognitive Assessment; SD: Standard Deviation; Wechsler Adult Intelligence Scale-III. ^1^Age and years of education as covariates. ^2^From group (interventions vs controls)-by-time (pre vs post) interaction. ^3^Effect size was taken as the value of eta squared. ^4^Δ Logical Memory was calculated by dividing Logical Memory II (delay) by Logical Memory I (immediate).

In the executive function tests, while there was no difference in TMT-A, there was a marginally significant interaction between group and time (F (1, 52) =2.83, p =0.098, η^2^ = 0.051) in TMT-B. A marginally significant interaction between group and time was also indicated in KPT (F (1, 54) =2.86, p =0.097, η^2^ = 0.049). Simple main effects showed that the score of the intervention group improved after the program completion (p =0.005). Significant differences in the scores were not seen in the verbal fluency tests, the MMSE and the MoCA-J.

### Intervention effects in MCI participants

**Figure 2:**
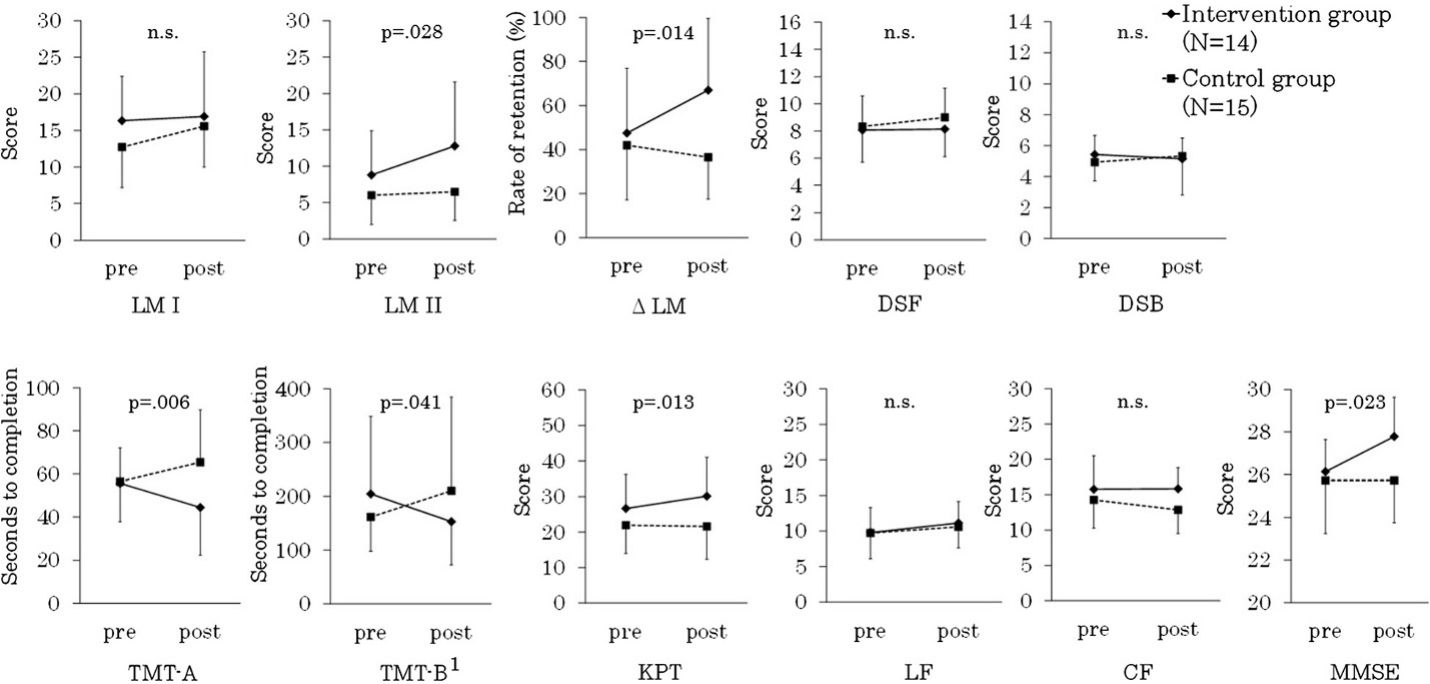
**Average scores of cognitive tests at pre and post program in participants with MCI.** MCI: Mild Cognitive Impairment; LM: Logical memory; ∆LM was calculated by dividing LM II by LM I; DSF: Digit Span forward; DSB: Digit Span backward; TMT-A: Trail Making Test part A; TMT-B: Trail Making test part B; KPT: Kana Pick-out Test; LF: Letter Fluency; CF: Category Fluency; MMSE: Mini Mental State Examination. ^1^Two cases which had a defect in inspection implementation were excluded from analysis. The criterion of MCI was participants who scored <26 in the Japanese version of the Montreal Cognitive Assessment at baseline measurement. The p values from group (interventions vs controls) – by – time (pre vs post) interaction.

In the intervention group, fourteen persons corresponded to the criterion of MCI. Fifteen persons fit this criterion in the control group. The scores of cognitive tests in each group of MCI are shown in Figure 2. Analysis of the intervention effects were conducted for MCI participants.

Significant interactions between group and time were seen such as in the analysis for full participants in LM II (F (1, 25) =5.42, p =0.028, η^2^ = 0.131) and Δ LM (F (1, 25) =7.05, p =0.014, η^2^ = 0.220). Simple main effects also showed an improvement in the intervention group after the program completion in LM II (p =0.002) and Δ LM (p =0.008). Although the ANCOVAs of LM were the same as analyses of all participants, the effect sizes were greater in MCI.

In MCI participants, the intervention effects were found both in executive function and attention tests. Significant interactions between group and time were seen in TMT-A (F (1, 25) =8.96, p =0.006, η^2^ = 0.257), TMT-B (F (1, 23) =4.68, p =0.041, η^2^ = 0.161), and KPT (F (1, 25) =7.18, p =0.013, η^2^ = 0.203). Simple main effects showed that the results of the intervention group improved after the program completion in TMT-A (p =0.029) and KPT (p =0.002).

Significant interaction between group and time was also seen in the MMSE (F (1, 25) =5.86, p =0.023, η^2^ = 0.187) and improvement in the score of the intervention group was found (p =0.003). In addition, in the global cognitive assessment, the intervention effects were as seen in MCI. There was no intervention effect in other cognitive tests.

## Discussion

The current study shows the effectiveness of cognitive intervention through a training program for picture book reading in community-dwelling older adults. In analyses of memory function, the intervention effect was obtained in the LM II which is an indicator of delayed verbal memory. Because the LM II is similar to everyday memory [23], this result is important, although there were no improvements in other types of memory. Learning new skills had good influence on the memory function [29].

Additional effect was obtained in the rate of memory retention for LM II. Since memory retention improved in LM, it is suggested that the participants in this program unconsciously developed memorization strategies. Since the intervention effect was not seen in DSF, DSB, and LM I which are the indices relevant to measurement of short-term memory, this program does not influence the increase in memory capacity. The participants have many opportunities to listen to, to memorize, and to tell a tale through picture book reading in the program. We speculated that repeating these activities might positively influence acquisition of memorization strategies for the participants.

Regarding executive function and attention tests, clear intervention effects were seen in the participants with MCI. The persons with MCI who participated in this program improved their executive functions of both verbal and operational tasks by performing complicated intellectual activities. These elements of the program acted on the global cognitive functions. In full analyses of participants, the clear intervention effect was not seen in the executive functional tests or global cognitive tests. For the participants for whom the global cognitive function is maintained, this program did not contribute to improvement in attention and executive function. However, the intervention effect was shown in MCI and so it can be said that this program contributes to maintenance of attention and execution function in older adults.

Extremely poor intervention effect was observed in the verbal fluency tests which were indicators of verbal function. Verbal function declines little with aging [7], but at the same time it is difficult to improve this function by short-term intervention. We can expect to improve verbal function by continuing such an intervention program over the long-term.

These findings suggest that the program aimed at learning and mastering methods of reading picture books contributes to preventing a decline of memory and executive function. Especially for MCI, it is effective to improve executive function and it might influence the maintenance of IADL. Based on neuroplasticity and cognitive reserve theory, cognitive abilities are expected to be modifiable at all stages of the life course [8]. The key to preventing cognitive decline may be to encourage older adults to engage in cognitively stimulating activities such as learning a new skill. Furthermore, long-term continuation of intellectual activities is also important to maintain cognitive functions.

The Advanced Cognitive Training for Independent and Vital Elderly (ACTIVE) study [30], which performed large-scale cognitive training interventions, has examined various kinds of health-related and economic variables. In the ACTIVE study, three cognitive training groups (memory, reasoning, and speed of processing) maintained their improvements for three years. The cognitive training groups have less functional decline in self-reported instrumental activity of daily living (IADL) as compared with control groups [31]. In a post-test conducted one-year after baseline comparison, intervening speed of processing significantly reduced subsequent annual predicted medical care expenditures [32]. Nevertheless, the cognitive training interventions did not generalize to cognitive functions other than the target [33].

One research project which focused on cognitive training using video games as an intervention program for improving intellectual activity reported an improvement in cognitive functions [34]. Cognitive training by playing video games for four weeks improved not only the video scores but also executive function and processing speed. However, it is difficult to continue the same cognitive training for many years. Also, the video game method did not involve communication with other people. Higher level of social engagement such as volunteer activity in old age is associated with better cognitive function [35–39]. Especially, initial findings of intergenerational volunteering suggest that a “real-world” intervention can be successful by integrating the individual effects of increased cognitive, social, and physical activity into daily life, thus allowing for large daily doses of stimulating activity [39].

Therefore, this study focused on senior volunteers who read picture books to children. Actually, most of the participants who mastered the skills of picture book reading through this program wish to continue the activity as active senior volunteers [40]. This program is valuable to encourage social contribution activities through engaging in cognitive training. Consciousness of social contribution and communication with children would also provide the motivation needed for participants to continue the activity.

This study has two limitations. First, as the present research was conducted in order to verify what kind of effect this program has on cognitive function, the cognitive functions were measured using many indicators despite the small sample. Further studies are necessary to verify the effectiveness of the program with a larger sample size. Second, it was challenging and somewhat troublesome to detect MCI using MoCA-J in the present study, although MoCA-J demonstrated excellent sensitivity and specificity in screening MCI. However, well-trained psychologists can objectively detect MCI and briefly assess sensitive change in cognitive function without a clinician. In fact, MCI is the transitional state between normal aging and dementia, and population-based studies conversely demonstrated that 14-40% of subjects with MCI experienced a so-called “reversion to normal” a year later [41, 42].

Extending an observation period might ensure lasting effects on IADL, in addition to cognitive function in this study, as ACTIVE study reported that cognitive training could prevent decline in self-reported IADL for ten years after intervention [43]. Therefore, an important future direction is to observe the long-term continuation effect of this program.

## Conclusions

We conducted a randomized controlled trial to explore the effectiveness of cognitive intervention through a training program for picture book reading in community-dwelling older adults. The intervention effect was seen in delayed verbal memory. Since the memory retention was improved, the possibility should be considered that the participants in this program learned the strategy of memorizing. For MCI participants, the intervention effects were clearly seen in memory, executive function and attention. These short-term findings suggest that this program contributed to prevent the decline in some domains of cognitive function.

## Authors’ information

HS has a PhD. MK has an ED (Educational Doctor). MY has a PhD. KN has a PhD. RS has a PT (Physical Therapist) and PhD. RT has a CP (Clinical Psychologist) and PhD. YM has a PhD. HO has a PSE (Psychiatric Social Worker) and MA. YF has a MD and PhD.

## Abbreviations

ANCOVA: Analysis of Covariance
DSB: Digit Span Backward
DSF: Digit Span Forward
GDS-15: Geriatric Depression Scale short form
IADL: Instrumental Activity of Daily Living
KPT: Kana Pick-out Test
LM: Logical Memory
MCI: Mild Cognitive Impairment
MMSE: Mini-Mental State Examination
MoCA-J: Japanese version of the Montreal Cognitive Assessment
TMIG: Tokyo Metropolitan Institute of Gerontology
TMT: Trail Making Test
WMS-R: Wechsler Memory Scale Revised
SD: Standard Deviation
TMIG-IC: Tokyo metropolitan institute of gerontology-Index of Competence.
